# Oncological Outcomes and Safety of Ovarian Preservation for Early Stage Adenocarcinoma of Cervix: A Systematic Review and Meta-Analysis

**DOI:** 10.3389/fonc.2019.00777

**Published:** 2019-08-14

**Authors:** Hongyan Cheng, Lanqing Huo, Liju Zong, Yujia Kong, Junjun Yang, Yang Xiang

**Affiliations:** Department of Obstetrics and Gynecology, Peking Union Medical College Hospital, Chinese Academy of Medical Sciences and Peking Union Medical College, Beijing, China

**Keywords:** cervical cancer, adenocarcinoma, ovarian preservation, outcome, risk factors

## Abstract

**Objectives:** To evaluate the oncological outcomes and safety of ovarian preservation, and to review the prognostic factors for ovarian metastases in early stage cervical adenocarcinoma.

**Methods:** PubMed, Embase, and Cochrane databases were searched for publications up to January 2019. Two investigators independently screened the studies for eligibility and extracted specific data. The primary outcomes were overall survival (OS) and progression-free survival (PFS). Pooled odds ratios (ORs) and 95% confidence intervals (CIs) were calculated using STATA statistical software version 19.0.

**Results:** A total of 68 unique manuscripts were identified through the search strategy, and 10 studies were included in the meta-analysis of the safety of ovarian preservation. Fixed-effects model was used because of moderate heterogeneity. Pooled results of the included studies showed that ovarian preservation is not associated with a statistically significant OS (OR 1.00, 95% CI 0.64–1.56, *I*^2^ = 25.7%) or PFS (OR 0.98, 95% CI 0.57–1.66, *I*^2^ = 0%) in early stage cervical adenocarcinoma. In addition, 19 studies were included in the review of prognostic factors for cervical adenocarcinoma and risk factors for ovarian metastases. The incidence of ovarian metastases was 0% in stage IA, 2.8% in stage IB, 3.4% in stage IIA, and 11.8% in stage IIB cervical adenocarcinoma. International Federation of Gynecology and Obstetrics (FIGO) stage, tumor size, deep stromal invasion (DSI), lymph node metastasis (LNM), and vaginal invasion were significantly related to poor prognosis. Risk factors associated with ovarian metastases included age, FIGO stage, tumor size, DSI, parametrial invasion, corpus uteri invasion, LNM, vaginal invasion, and blood vessel invasion.

**Conclusions:** Ovarian preservation in young patients with early stage cervical adenocarcinoma is safe and has no significant effect on OS or PFS. Preserving ovaries in patients with FIGO stage IIB seems not reasonable because of the high rate of ovarian metastasis.

## Introduction

Cervical cancer incidence has been declining for the past several decades worldwide because of the successful implementation of screening programs (1, 2). However, the proportion of young patients with early stage cervical cancer, especially adenocarcinoma, is increasing greatly (3). According to the report of the American Cancer Society (4), cervical cancer continues to be the second leading cause of cancer death in women aged 20–39 years (nine deaths per week were recorded in this age group). Adenocarcinoma accounts for ~28% of all cervical cancer cases (5). Adenocarcinoma in cervical cancer even reached to 40% in women aged ≤25 years in a recently published study (6). Conservation of ovarian endocrine function or fertility sparing is greatly desirable in this group of young patients.

Unlike squamous cell carcinoma (SCC), cervical adenocarcinoma is believed to be more aggressive and may have an inclination of blood vessel invasion, deep stromal invasion (DSI), and lymph node metastases (LNM) (7). Nevertheless, a recent study showed that early stage cervical adenocarcinoma has a good prognosis, and the 5-year survival rate is >80% (8). In the study of Kasamatsu et al. (9), no significant difference in survival or relapse between SCC and adenocarcinoma was found.

Ovarian preservation in early stage SCC has been well-established since McCall et al. (10) firstly presented it in 1958. However, no consensus about the safety of ovarian preservation in cervical adenocarcinoma exists. Studies showed that the incidence of ovarian metastases in early stage adenocarcinoma is higher than that in SCC, but mostly lower than 5% (11–15), and a few studies reported slightly high, which were 10.2% (16) and 12.9% (17). Radical bilateral salpingo-oophorectomy sacrifices endocrine function while possibly eliminating the concealed lesions in the ovaries. Young patients experience menopausal symptoms including immediate hot flashes, vaginal atrophy, osteoporosis, and emotional problems, earlier than expected (18). Performing the least aggressive procedure without sacrificing oncologic safety is vital for young women diagnosed with early stage cervical adenocarcinoma.

In this study, we systematically reviewed all available relevant studies and conducted a meta-analysis to evaluate the oncological outcomes and safety of ovarian preservation. In addition, we summarized the prognostic factors for cervical adenocarcinoma and risk factors for ovarian metastases.

## Materials and Methods

### Search Strategy

PubMed, Embase, and Cochranes database were searched for publications up to January 2019. We used the following search terms in the title or abstract: “cervical neoplasm,” “adenocarcinoma,” “ovarian preservation,” and “ovarian conservation.” Both free words and Emtree terms were applied in the search. The language was limited to “English” and the object to “human” (Supplementary Data Sheet 1).

### Inclusion and Exclusion Criteria

Studies were included in this meta-analysis if: (1) the diagnosis of cervical adenocarcinoma based on International Federation of Gynecology and Obstetrics (FIGO) stage I or II adenocarcinoma of cervix; (2) they were prospective, retrospective cohort, or cross-sectional original studies; (3) they included at least 10 patients; (4) at least one outcome, such as overall survival (OS) or progression-free survival (PFS) was assessed; (5) the odds ratios (ORs) and their 95% confidence intervals (95% CIs), or the number of events used to calculate them was reported.

The inclusion criteria for the review of prognostic factors for cervical adenocarcinoma and risk factors for ovarian metastases were as follows: (1) original studies that reported the ovarian metastasis rate of FIGO stage I or II cervical adenocarcinoma; (2) studies that evaluated the prognostic factors for cervical adenocarcinoma or risk factors for ovarian metastases using a statistical analysis.

Studies were excluded if they meet following criteria: (1) review articles or case reports with fewer than 10 cases; (2) lack of sufficient data to estimate OR and 95% CI; (3) reporting duplicate or overlapping data; (4) without full text.

### Data Extraction

The following information was extracted from each eligible study: first author's name, published year, study design, country, patients' mean age, FIGO stage, number of patients, number of patients who underwent hysterectomy and oophorectomy/ovarian preservation, incidence of ovarian metastases, and data on OS and/or PFS. Two investigators (CHY and ZLJ) extracted the data independently, and any discrepancies and disagreements were discussed and resolved by the adjudicating senior author (YJJ).

### Quality Assessment

The Newcastle-Ottawa Quality Assessment Scale for case-control studies was used to evaluate the included studies. Selection, comparability, and exposure were measured. A maximum of nine stars was assigned to each study: 4 for selection, 2 for comparability, and 3 for exposure. A final score > 6 was considered as a high quality (19, 20). Two authors (KYJ and HLQ) independently assessed the quality of the included studies and disagreements were resolved by discussion (Supplementary Table 1).

### Statistical Analysis

Survival data, including OS, PFS, and time-to-event were calculated as dichotomous data. STATA statistical software version 19.0 (Stata Corp. LLC, College Station, TX, USA) was used to pool the study-specific ORs and 95% CIs and generate forest plots. Cochran's-Q test and *I*^2^ statistics were used to evaluate heterogeneity (21). Heterogeneity was considered significant when the *P*-value <0.05 in Cochran's-Q test and when *I*^2^ > 50% in *I*^2^ statistics. If so, random-effects model was used. Otherwise, a fixed-effects model was used. Publication bias was evaluated by funnel plots. Sensitivity analysis was performed by omitting one study at a time to assess its effect on the final result.

## Results

### Search Results and Study Characteristics

**Figure 1:**
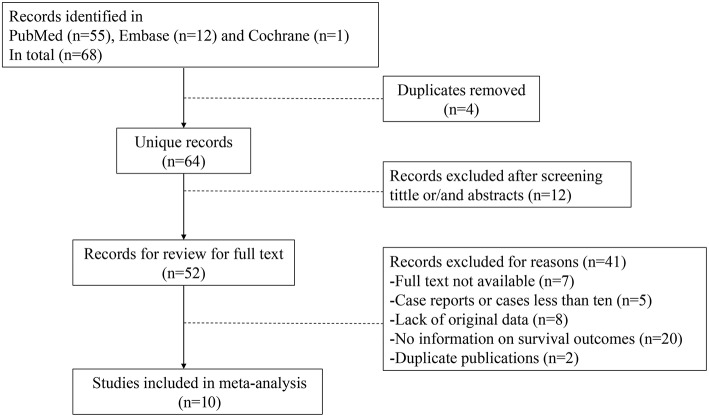
Flowchart of screening process.

In total, 68 unique manuscripts were identified through the search strategy, and 10 studies were included in the meta-analysis of the safety of ovarian preservation. The reasons for excluding records are depicted in Figure 1. A total of 19 studies were included in the review of prognostic factors for cervical adenocarcinoma and risk factors for ovarian metastases based on the inclusion and exclusion criteria. The included studies were all retrospective in nature, and detailed characteristics of the 10 studies are presented in Table 1 (7, 9, 12, 13, 22–28).

**Table 1:** Characteristics of studies included in the meta-analysis.

**Author**	**Year**	**Study period**	**Country**	**Mean age (Y)**	**FIGO stage**	**No. patients**	**Ovarian preservation (*n*)**	**Oophorectomy (*n*)**	**Rate of ovarian metastases**	**Survival outcome reported**
Hopkins et al. (22)	1987	1970–1984	US	NA	I	24	8	16	0/16	OS
Angel et al. (12)	1992	1966–1990	US	47	I	59	41	18	0/41	OS, PFS
Sutton et al. (13)	1992	1981–1984	GOG	NA	I	121	41	80	2/80 (2.5%)	PFS
Kasamatsu et al. (9)	2009	1984–2003	Japan	48	I-II	123	22	100	6/100 (6%)	OS, PFS
Chen et al. (7)	2016	1999–2013	China	43.6	I-II	194	33	153	5/153 (3.3%)	OS, PFS
Ruengkhachorn et al. (23)	2016	2006–2013	Thailand	44.9	I	35	16	19	0/19	PFS
Matsuo et al. (24)	2017	1983–2012	SEER	45.3	I	4,019	960	3,059	NA	OS
Hu et al. (25)	2017	1994–2015	China	46.2	I–II	105	19	86	3/86 (3.5%)	OS
Xie et al. (26)	2018	2003–2015	China	44.3	I–II	128	15	113	1/113 (0.9%)	OS
Guo et al. (27)	2018	1995–2017	China	NA	I–II	267	44	223	13/223 (5.8%)	PFS
Total	–	–	–	45.6	–	5,075	1,199	3,867	30/831 (3.61%)	–

*FIGO, International Federation of Gynecology and Obstetrics; GOG, Gynecologic Oncology Group; NA, not available; OS, overall survival; PFS, progression free survival*.

### Oncological Outcomes

**Figure 2:**
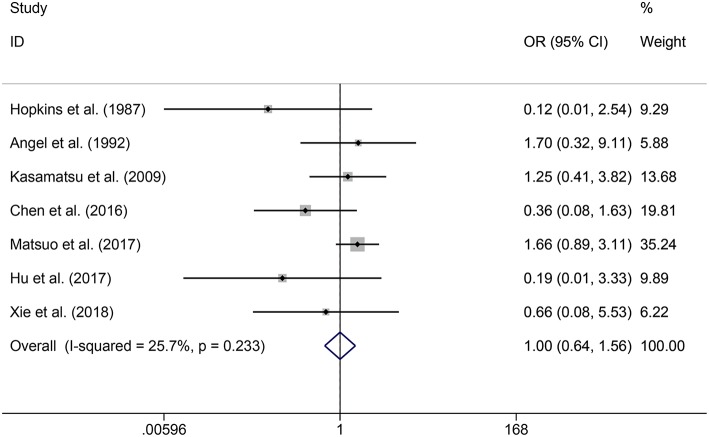
Forest plots of OS for ovarian preservation vs. oophorectomy in early stage cervical adenocarcinoma. Weights were from fixed-effects model.

**Figure 3:**
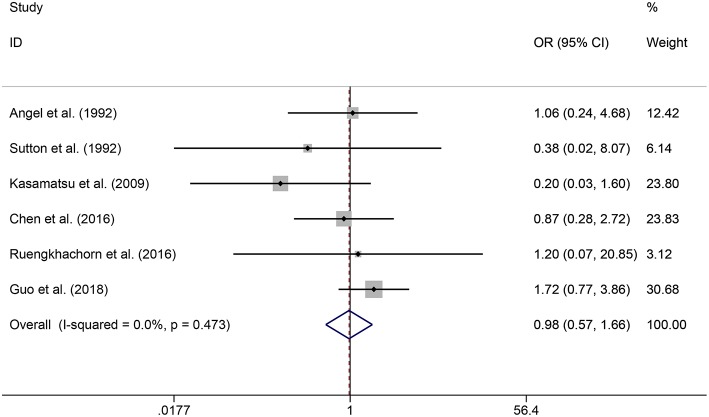
Forest plots of PFS for ovarian preservation vs. oophorectomy in early stage cervical adenocarcinoma. Weights were from fixed-effects model.

**Figure 4:**
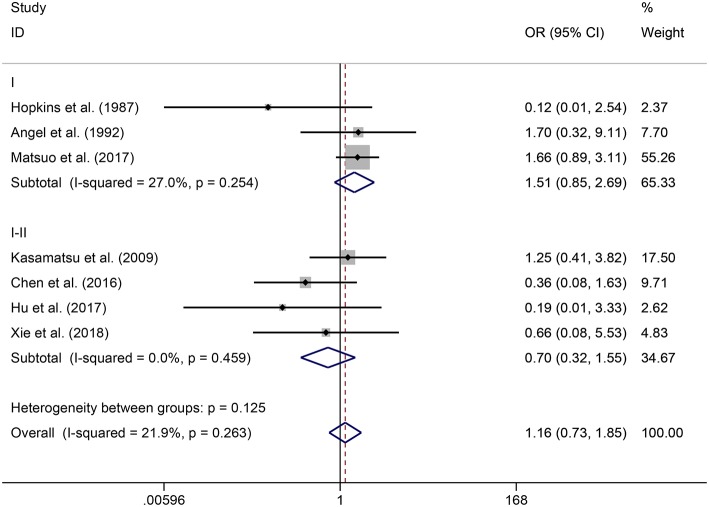
Subgroup analysis of OS for ovarian preservation vs. oophorectomy in early stage cervical adenocarcinoma. Weights were from fixed-effects model.

**Figure 5:**
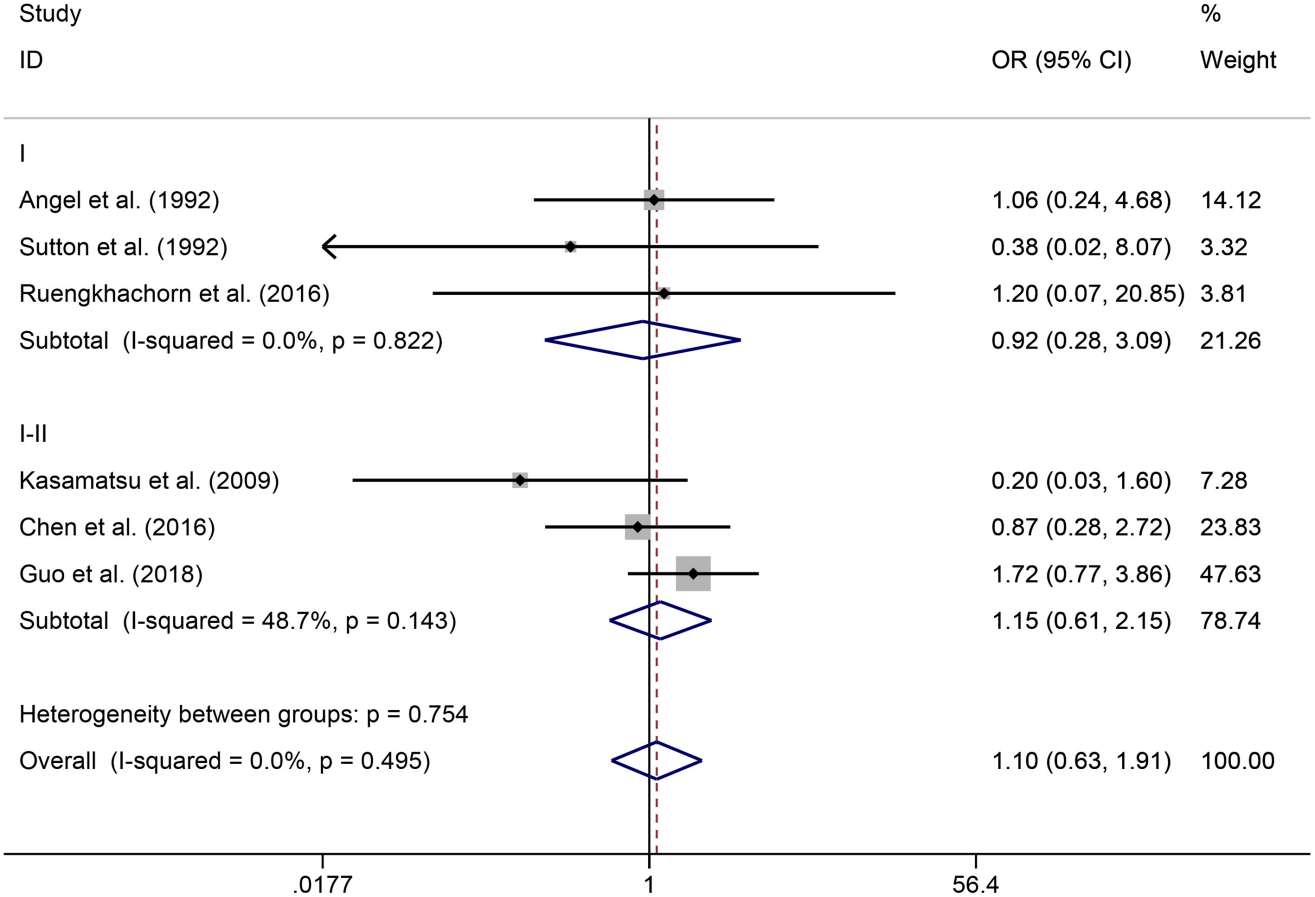
Subgroup analysis of PFS for ovarian preservation vs. oophorectomy in early stage cervical adenocarcinoma. Weights were from fixed-effects model.

**Figure 6:**
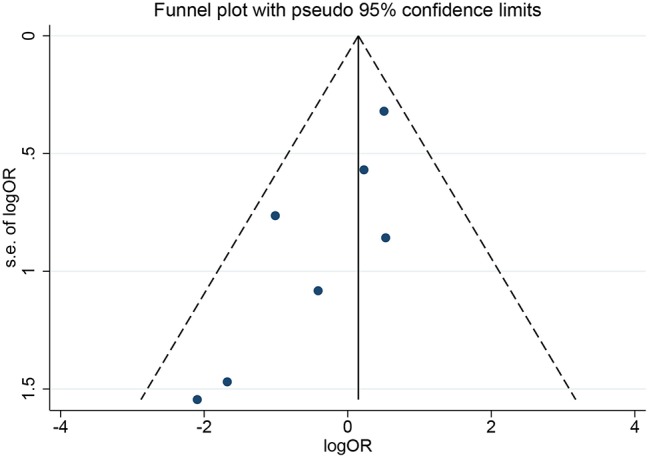
Funnel plots of OS show a low risk of publication bias.

**Figure 7:**
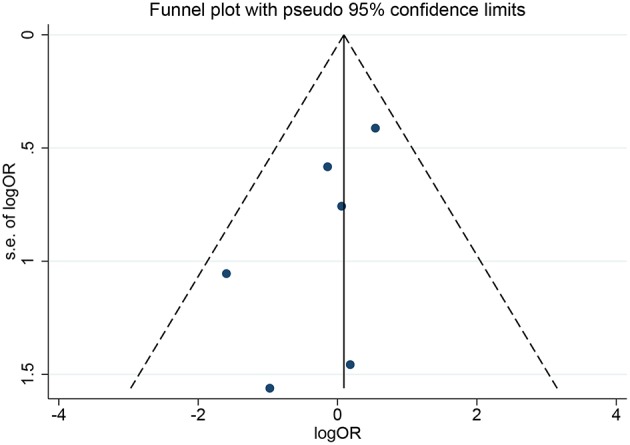
Funnel plots of PFS show a low risk of publication bias.

**Figure 8:**
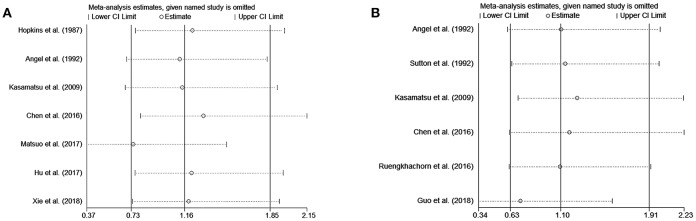
Sensitivity analysis of OS **(A)** and PFS **(B)** show that no significant changes in the final result after each study was omitted sequentially.

In the meta-analysis, no heterogeneity in OS and PFS among the studies was found, thus, a fixed-effects model was used. Based on the pooled results from the included studies, ovarian preservation is not associated with a statistically significant OS (OR 1.00, 95% CI 0.64–1.56, *I*^2^ = 25.7%; Figure 2) or PFS (OR 0.98, 95% CI 0.57–1.66, *I*^2^ = 0%; Figure 3) in early stage cervical adenocarcinoma. Subgroup analysis (Figures 4, 5) and funnel plot results (Figures 6, 7) showed that our study has a low risk of publication bias. No significant changes in the final result, after each study was omitted sequentially, were observed (Figure 8).

### Prognostic Factors for Cervical Adenocarcinoma and Risk Factors for Ovarian Metastases

Table 2 shows the results of the literature review. The incidence of ovarian metastases was 0% in stage IA, 2.8% in stage IB, 3.4% in stage IIA, and 11.8% in stage IIB cervical adenocarcinoma. Five studies (7, 9, 12, 22, 26) showed that FIGO stage, tumor size, DSI, LNM, and vaginal invasion are significantly related to poor prognosis. Nine studies (7, 14, 16, 17, 29–34) reported that age, FIGO stage, tumor size, DSI, parametrial invasion (PMI), corpus uteri invasion (CUI), LNM, vaginal invasion, and blood vessel invasion are significantly associated with ovarian metastases.

**Table 2:** Overview of prognostic factors for cervical adenocarcinoma and risk factors for ovarian metastases reported in the studies.

**Author**	**Year**	**No. of patients**	**Stage**	**Rate of ovarian metastases**	**Variables included in multivariate analysis**								
					**Age**	**FIGO stage**	**Tumor size**	**Deep stromal invasion**	**Parametrial invasion**	**Corpus uteri invasion**	**Lymph node metastasis**	**Vaginal invasion**	**Blood vessel invasion**
Xie et al. (26)	2018	128	IA-IIB	1/113 (0.9%)		^*^					^*^		
Kasamatsu et al. (9)	2009	123	IB	1/87 (1.15%)			^*^				^*^	^*^	
			IIA	0									
			IIB	3/22 (13.6%)									
Angel et al. (12)	1992	59	I	0/41							^*^		
Hopkins et al. (22)	1987	24	I	0/16							^*^		
Chen et al. (7)	2016	194	IA	0/9			^*^	^*^ ^#^	^#^		^*^ ^#^		
			IB	2/100 (2%)									
			IIA	2/26 (7.7%)									
			IIB	1/18 (5.6%)									
Nakanishi et al. (29)	2000	240	IA	0/15					^#^		^#^		
			IB	7/178 (3.9%)									
			IIA	0/11									
Hu et al. (30)	2013	183	IB	1/130 (0.8%)		^#^	^#^		^#^	^#^			
			IIA	3/39 (7.7%)									
			IIB	1/14 (7.1%)									
Natsume et al. (17)	1999	62	IB	1/31 (3.2%)				^#^			^#^		
			IIA	1/3 (33.3%)									
			IIB	6/28 (21.4%)									
Shimada et al. (31)	2006	546	IB	14/376 (3.7%)					^#^				
			IIA	2/38 (5.3%)									
			IIB	13/132 (9.8%)									
Zhou et al. (32)	2017	312	IA	0/9					^#^	^#^		^#^	
			IB	5/217 (2.3%)									
			IIA	8/74 (10.8%)									
			IIB	1/12 (8.3%)									
Yamamoto et al. (16)	2001	89	IB	1/50 (2%)									^#^
			IIA	0/2									
			IIB	6/37 (16.2%)									
Landoni et al. (14)	2007	380	IA-IIA	9/380 (2.4%)	^#^	^#^		^#^					
Lu et al. (33)	2016	101	IA	0/1						^#^			
			IB	4/88 (4.6%)									
			IIA	1/12 (8.3%)									
Toki et al. (11)	1991	36	IB-IIB	2/36 (5.6%)									
Tabata et al. (34)	1987	48	IB	2/26 (7.7%)									
			IIA	0/2									
			IIB	2/13 (15.4%)									
Kjorstad et al. (15)	1984	150	IB	2/150 (1.3%)									
Guo et al. (27)	2018	267	I-II	13/223 (5.8%)									
Sutton et al. (13)	1992	121	IB	2/80 (2.5%)									
Ruengkhachorn et al. (23)	2016	35	IA	0/19									
Total		3,098		OM:		IA 0/53		IB 42/1513 (2.8%)		IIA 17/208 (3.4%)		IIB 30/254 (11.8%)	

*No., number; FIGO, International Federation of Gynecology and Obstetrics*.

* *Prognostic factors for cervical adenocarcinoma*.

# *Risk factors for ovarian metastases*.

## Discussion

In this review and meta-analysis on the prognostic significance of ovarian preservation in early stage cervical adenocarcinoma, we found that ovarian preservation is not associated with a statistically significant OS or PFS in early stage cervical adenocarcinoma. Ovarian preservation has no adverse effect on the prognosis in early stage cervical adenocarcinoma (7, 24, 28). Moreover, the overall incidence of ovarian metastases is 0% in stage IA, 2.8% in stage IB, 3.4% in stage IIA, and 11.8% in stage IIB cervical adenocarcinoma, which are extremely low except that in stage IIB disease. Although some studies (11, 14, 17) proved that ovarian metastases are more common in cervical adenocarcinoma than in SCC, patients with early stage adenocarcinoma or SCC who underwent radical hysterectomy have a similar prognosis and spread pattern according to the study of Kasamatsu et al. (18). A consensus that ovarian preservation is safe in stage IA cervical adenocarcinoma was reached because the rate of ovarian metastases was 0% in numerous studies (7, 23, 29, 32, 33). In addition, ovarian preservation also appears safe in patients with cervical adenocarcinoma that is earlier than stage IIA because ovarian metastases are rare (2.8% in stage IB, and 3.4% in stage IIA in our review). Furthermore, previous studies reported no significant difference in OS after ovarian preservation among patients with SCC and adenocarcinoma whose disease stage is earlier than stage IIA (13, 15). Notably, ovarian preservation must be performed carefully in stages IB and IIA because studies showed that tumor size >4 cm is related to a poorer prognosis (7, 9). For stage IIB cervical adenocarcinoma, ovarian preservation is inappropriate because of a high risk of ovarian metastases (11.8% in this review). These cases probably accompanied with other factors that are related to poor prognosis, included LNM, CUI, PMI, and DSI (7, 9).

The FIGO clinical staging system of cervical cancer has been constantly updated. Imaging and pathology have been recently used to supplement clinical findings with respect to tumor size and extent (35). The most obvious change in the different versions of the staging system are related to tumor size (≤2 cm, 2–4 cm, and >4 cm), which could be because numerous studies showed that tumor size is an independent prognostic factor for OS in cervical cancer (7, 9, 36). In the retrospective study and meta—analysis of Hu et al. (30), they suggested that tumor size >4 cm are associated with ovary metastasis. Notably, according to the latest 2018 FIGO staging system, the risk in cervical cancer mortality in stage IB2 disease increased by nearly 2-fold compared to that in IB1 disease, which suggests that identifying the tumor size (i.e., ≥2 or <2 cm) is necessary when deciding whether to preserve ovaries or not (35).

For many years, ovaries were sacrificed in radical surgery for cervical cancer. However, there has been increasing awareness of the value of retaining the ovaries maintain a sense of well-being among young women. Premenopausal castration could cause immediate menopause, early hot flashes, and vaginal atrophy, as well as a number of long-term consequences, including an increased risk of cardiovascular disease, osteoporosis, hip fracture, Alzheimer's disease, and emotional problems (37). Hence, patients would need long-term menopause hormonal therapy (MHT) to alleviate the symptoms, let alone the poor compliance and high expense of MHT (38). Maintenance of ovarian function is beneficial to the physiologic and psychosexual health of young patients without significantly increasing their risk of relapse.

Another concern of ovarian preservation is its safety. In our review, the incidence of ovarian metastases is extremely low in patients who underwent oophorectomy, except that in stage IIB cervical adenocarcinoma. A study of Greer et al. (39) including 45 patients with stage IB cervical adenocarcinoma who had ovarian conservation showed that none of the patients with recurrence have ovarian involvement. Ranney et al. (40) conducted a study of 2,132 patients who underwent hysterectomy (1,557 of the patients had their ovarian tissue retained) and suggested that the incidence of primary ovarian cancer following a hysterectomy is ~0.2%. Moreover, pelvic radiation therapy is often indicated in patients after surgery. However, it may result in premature ovarian insufficiency (POI). One of the options to prevent POI is ovarian transposition, in which the ovaries are placed outside the radiation field thereby reducing the exposure to radiation and total dose of irradiation. A recent review demonstrated that the ovarian survival after ovarian transposition ranges from 63.6 to 100% (41).

Furthermore, our results showed that oophorectomy has no prognostic benefit in early stage cervical adenocarcinoma. Studies demonstrated that all patients with ovarian metastases have at least one of the following risk factors: large tumor size, DSI, positive lymph node, and vaginal invasion (12). Ovarian metastases in cervical adenocarcinoma are more likely visible and present in both ovaries (14). Although the rate of ovarian metastases in cervical adenocarcinoma is slightly higher than that in SCC, no difference in nodal metastases, recurrence, or OS between the two histologic subtypes was observed (9, 12, 13, 29).

On the contrary, Shimada et al. (31) demonstrated that the outcomes of patients with ovarian metastases are extremely poor and not related to FIGO stage and histological type. Landoni et al. (14) retrospectively analyzed 380 patients with stage IA2-IIA cervical adenocarcinoma and found that the incidence of ovarian metastases was 2.3%; they suggested that oophorectomy be performed in all patients with adenocarcinoma. Balancing the risk and benefit of ovarian preservation is crucial for gynecologists. Thus, some researchers summarized the following selection criteria for ovarian preservation in patients with cervical adenocarcinoma: age < 45 years, stage < IB, tumor size < 4 cm, no DSI, no PMI, no CUI, no LNM (MRI, CT-scan, or PET-scan), and no lymphatic vascular space invasion (33, 42). In our study, we found that FIGO stage, tumor size, DSI, LNM, and vaginal invasion are significantly related to poor prognosis in cervical adenocarcinoma. In addition, the following risk factors were significantly related to ovarian metastases: age, FIGO stage, tumor size, DSI, PMI, CUI, LNM, vaginal invasion, and blood vessel invasion. Hopefully, which could potentially provide reference for clinical decision. Gynecologists should meticulously examine adjacent organs, intra-operative specimen opening, and frozen section in suspicious cases before making a decision on whether to perform ovarian preservation or not.

This study has several limitations. Firstly, the long time span among included studies might cause bias because the staging system is updating with time. Second, the nature of retrospective chart reviews is undeniable. Lastly, different methodologies were used in the included studies, which may cause heterogeneity. Nevertheless, our result suggests that ovarian preservation is not associated with a statistically significant OS or PFS in early stage cervical adenocarcinoma. Further prospective and randomized trials are required to validate our findings.

## Conclusions

Ovarian preservation in young patients with early stage cervical adenocarcinoma is safe and has no significant effect on OS or PFS. Preserving ovaries in patients with FIGO stage IIB seems not reasonable because of the high rate of ovarian metastasis.

## Author Contributions

HC, YX, JY, LZ, YK, and LH: study conception and design and manuscript review. HC, LZ, and JY: literature review and data extraction. YK and LH: quality control. HC and YK: statistical analysis. HC and LZ: manuscript preparation.

### Conflict of Interest Statement

The authors declare that the research was conducted in the absence of any commercial or financial relationships that could be construed as a potential conflict of interest.

## Supplementary Material

Supplementary Data Sheet 1 Search strategy detail.

Supplementary Table 1 Quality assessment of included studies.
