# Supplementary material for: Oncological Outcomes and Safety of Ovarian Preservation for Early Stage Adenocarcinoma of Cervix: A Systematic Review and Meta-Analysis
Source: Front Oncol. 2019 Aug 14;9:777. doi: 10.3389/fonc.2019.00777 (PMC6702263; doi:10.3389/fonc.2019.00777)
Supplement: Supplementary Data Sheet 1 — Search strategy detail. [file Data_Sheet_1.docx]

Search from PubMed:

(((Adenocarcinoma) AND (((((((((((((((((((((((((((Cervical Neoplasm, Uterine[All Fields]) OR Cervical Neoplasms, Uterine[All Fields]) OR Neoplasm, Uterine Cervical[All Fields]) OR Neoplasms, Uterine Cervical[All Fields]) OR Uterine Cervical Neoplasm[All Fields]) OR Neoplasms, Cervical[All Fields]) OR Cervical Neoplasms[All Fields]) OR Cervical Neoplasm[All Fields]) OR Neoplasm, Cervical[All Fields]) OR Neoplasms, Cervix[All Fields]) OR Cervix Neoplasms[All Fields]) OR Cervix Neoplasm[All Fields]) OR Neoplasm, Cervix[All Fields]) OR Cancer of the Uterine Cervix[All Fields]) OR Cancer of the Cervix[All Fields]) OR Cervical Cancer[All Fields]) OR Uterine Cervical Cancer[All Fields]) OR Cancer, Uterine Cervical[All Fields]) OR Cancers, Uterine Cervical[All Fields]) OR Cervical Cancer, Uterine[All Fields]) OR Cervical Cancers, Uterine[All Fields]) OR Uterine Cervical Cancers[All Fields]) OR Cancer of Cervix[All Fields]) OR Cervix Cancer[All Fields]) OR Cancer, Cervix[All Fields]) OR Cancers, Cervix[All Fields]) OR Uterine Cervical Neoplasms[MeSH Terms]))) AND ((ovarian preservation) OR ovarian conservation)

Search from Embase:

#1 AND #4 12

#4：#2 OR #3 918

#3 'ovarian conservation' 279

#2 'ovarian preservation' 671

#1 'uterine cervix adenocarcinoma'/exp
