# Supplementary material for: Oncological Outcomes and Safety of Ovarian Preservation for Early Stage Adenocarcinoma of Cervix: A Systematic Review and Meta-Analysis
Source: Front Oncol. 2019 Aug 14;9:777. doi: 10.3389/fonc.2019.00777 (PMC6702263; doi:10.3389/fonc.2019.00777)
Supplement: Supplementary Table 1 — Quality assessment of included studies. [file Table_1.docx]

Supplementary Table 1. Quality assessment of included studies

|  | Hopkins et al. | Angel et al. | Sutton et al. | Kasamatsu et al. | Chen et al. | Ruengkhachorn et al. | Matsuo et al. | Hu et al. | Xie et al. | Guo et al. |
| --- | --- | --- | --- | --- | --- | --- | --- | --- | --- | --- |
| **Selection** |  |  |  |  |  |  |  |  |  |  |
| Case definition with independent validation | 1 | 1 | 1 | 1 | 1 | 1 | 1 | 1 | 1 | 1 |
| Consecutive or obviously representative series of cases | 1 | 1 | 1 | 1 | 1 | 1 | 1 | 1 | 1 | 1 |
| Community controls | 1 | 1 | 1 | 1 | 1 | 1 | 1 | 1 | 1 | 1 |
| No endpoint of disease in controls at start study | 1 | 1 | 1 | 1 | 1 | 1 | 1 | 1 | 1 | 1 |
| **Comparability** |  |  |  |  |  |  |  |  |  |  |
| Study controls for age | 0 | 1 | 0 | 1 | 1 | 0 | 1 | 1 | 0 | 0 |
| Study controls for FIGO stage | 0 | 0 | 0 | 1 | 1 | 0 | 0 | 1 | 0 | 0 |
| **Exposure** |  |  |  |  |  |  |  |  |  |  |
| Ascertainment of exposure from secure record | 1 | 1 | 1 | 1 | 1 | 1 | 1 | 1 | 1 | 1 |
| Same method and ascertainment for cases and controls | 1 | 1 | 1 | 1 | 1 | 1 | 1 | 1 | 1 | 1 |
| Same non-response rate for both groups | 1 | 1 | 1 | 1 | 1 | 1 | 1 | 1 | 1 | 0 |
| **Total score** | 7 | 8 | 7 | 9 | 9 | 7 | 8 | 9 | 7 | 6 |
